# Changes in patterns of use and perceptions of cannabis among students in Canada: A decade of data from the Canadian Student Alcohol and Drugs Survey

**DOI:** 10.1016/j.dadr.2025.100399

**Published:** 2025-11-25

**Authors:** Samantha Goodman, Sebastian A. Srugo, Emilia Krzeminska, Hanan Abramovici

**Affiliations:** a Office of Cannabis Science and Surveillance, Strategic Policy Directorate, Controlled Substances and Cannabis Branch, Health Canada, Ottawa, ON, Canada; b Office of Drug Research and Surveillance, Controlled Substances and Overdose Response Directorate, Controlled Substances and Cannabis Branch, Health Canada, Ottawa, ON, Canada

**Keywords:** Cannabis, Adolescents, Surveillance

## Abstract

**Introduction:**

The Canadian Student Alcohol and Drugs Survey is a biennial, repeat cross-sectional survey of grade 7–12 students in the Canadian provinces. This study examined cannabis-related behaviours at five timepoints before and after legalization of cannabis for non-medical purposes.

**Methods:**

Trends over time were examined using data from 2014–15 to 2023–24 (n = 264,558). Binary logistic regression examined changes in cannabis use and related behaviours, including frequency; usual method of use; perceived risk and access; usual source; and motor vehicle behaviours. Data were stratified by sex and grade group (grade 7–9 vs. 10–12).

**Results:**

Overall, there was no change in prevalence of past 12-month, past 30-day, or frequent cannabis use (p > 0.05 for all); however, modest increases were observed among females and grade 7–9 students (p < 0.05 for both). Vaping surpassed smoking as the most common method of consumption in 2023–24. Smoking and dabbing cannabis decreased over time, whereas vaping and eating cannabis increased (p < 0.001 for all). Perceived risk of regularly smoking cannabis decreased (p < 0.001), and perceived ease of cannabis access increased (p < 0.001). The most common cannabis sources were social sources. There was no change in driving after using cannabis (p > 0.05), whereas there was a recent increase in riding with a driver who had used cannabis (p < 0.001).

**Conclusion:**

While legalization and regulation of cannabis for non-medical purposes was not associated with increases in overall cannabis use among students in Canada, increasing rates of use in females and younger students, and changes in perceptions of risk and accessibility require continued monitoring.

## Highlights

• Data are from grade 7–12 students in the Canadian Student Alcohol and Drugs Survey.
• Over time, cannabis use increased only in grade 7–9 and female students.
• Over time, students perceived cannabis to be less risky and more accessible.
• In 2023–24, 72 % of students who consumed cannabis reported vaping it.
• Patterns of use, perceived risk and access require continued monitoring.

## 1 Introduction

In October 2018, Canada legalized and regulated cannabis for non-medical purposes through the *Cannabis Act* (Government of Canada, 2019a). During Phase I of legalization and regulation, only dried cannabis, plants, seeds, and oils for oral consumption were available for legal retail sale. In October 2019, Phase II of legalization and regulation allowed the legal retail sale of cannabis edibles, beverages, extracts and topical products (Government of Canada, 2019b).

One of the objectives of the *Cannabis Act* is to protect the health and safety of Canadians, including keeping cannabis out of the hands of youth (Government of Canada, 2019c). Young people are particularly susceptible to the effects of tetrahydrocannabinol (THC), the primary psychoactive constituent in cannabis, as the brain continues to develop until approximately age 25. Research has demonstrated that early cannabis use can harm the developing brain, impairing memory, learning, attention, and decision making, and increasing the risk of psychotic symptoms and cannabis use disorder (Bagot et al., 2015; Centers for Disease Control and Prevention (CDC), 2024; Hamaoui et al., 2025). To partly address this objective, Canada set the minimum legal age for obtaining cannabis at 18 years; this was increased to 19 years in all provinces and territories but Alberta (18 years) and Quebec (21 years, as of January 2020). There are strict penalties for selling cannabis to youth, and packaging or labelling cannabis in a way that is appealing to youth and promoting cannabis to young people are prohibited (Government of Canada, 2021a).

These measures are important, as Canada had the highest rates of past 30-day cannabis use among 43 countries according to the 2022 Health Behaviour in School-Aged Children study (HBSC) (World Health Organization (WHO), 2023). Canadian surveys of adolescents aged 12–19 years or students in grades 7–12 suggest that approximately 11 %-18 % of Canadian youth have consumed cannabis in the previous year, 7 %-12 % have used in the previous 30 days, and 2 %-3 % use daily/almost daily (Centre for Addiction and Mental Health (CAMH), 2023b; Health Canada, 2025a; University of Waterloo, 2025a, University of Waterloo, 2025b). Frequent (i.e., weekly or daily) cannabis use is associated with greater risk of physical and mental health concerns and is important to measure, along with detailed information on product use, since cannabis products vary widely and carry different health risks (Government of Canada, 2021b).

However, few studies provide data on detailed patterns of cannabis use among large samples of youth. HBSC only reports on prevalence of and age of initiation to cannabis use; since 2023, the Canadian Community Health Survey (CCHS) has only surveyed adults; and the Canadian Cannabis Survey (CCS) does not survey youth under 16 years (approximately grade 10) (Government of Canada, 2024a, Statistics Canada, 2024; WHO, 2023). Moreover, recent cycles of CCHS and CCS had relatively modest samples of youth reporting past 12-month cannabis use (approx. 300–500) (Government of Canada 2024b, Health Canada, 2025a), making it difficult to stratify estimates among youth who use cannabis.

The Canadian Student Alcohol and Drugs Survey (CSADS), formerly the Canadian Student Tobacco, Alcohol and Drugs Survey, is a biennial, school-based survey that has collected information on drug use among students since 1994 (Health Canada, 2025b; University of Waterloo, n.d). CSADS collects data from tens of thousands of students in grades 7–12, representing approximately two million students in Canada per cycle. This generates much larger samples of youth who use cannabis (close to 10,000 in 2023–24), making it a key survey for estimating cannabis use among youth (Health Canada, 2025b). The primary objective of the current study was to analyze trends over time in cannabis use and patterns of use among students, including by sex and grade group, leading up to and following legalization of cannabis for non-medical purposes in Canada. A secondary objective was to examine trends in cannabis indicators, including perceived risk of regular cannabis use and perceived accessibility of cannabis over the same time period.

Prior to 2014, data were collected as part of the Youth Smoking Survey, which initially only asked one item on cannabis use and did not survey students above grade 9 until 2006 (Statistics Canada, 2007). To ensure comparability across cycles, the current study used five cycles of CSADS data beginning in 2014–15. Health Canada has recently published key findings and an interactive data tool for CSADS online (Health Canada, 2025b, Health Canada, 2025c). This paper extends this work by offering more granular insights and using statistical models to compare cannabis indicators over a decade, with two timepoints pre-Phase I of cannabis legalization^1^ (2014–15 and 2016–17), one peri-legalization timepoint (2018–19), and two timepoints post-Phase II of legalization (2021–22 and 2023–24).

## 2 Methods

Respondents were 264,558 students in grades 7–12^2^ from all participating provinces.^3^ Students living in the territories and those attending specialized schools or schools with small class sizes (averaging <18–20 students) were excluded.

To obtain the student sample, a stratified single-stage cluster design was used. Schools were selected from strata and asked to participate; all eligible students within participating schools were surveyed. Publicly available user guides provide detailed information, including strata selection and province-specific methodology, imputation of missing data, and participation rates at the school board, school and student levels (Health Canada, 2025d). From 2014–15 to 2023–24, completion rates among eligible students ranged from 60 % to 76 %. The project was approved by the Health Canada-Public Health Agency of Canada Research Ethics Board (REB# 2009–0060 and 2020–040H), and ethics committees at the school-board level where applicable.

### 2.1 Measures

Students who participated in the survey provided detailed information on sociodemographic characteristics and cannabis use (including frequency of use), products used, usual cannabis source, perceived risk of regular cannabis use, perceived ease of access, age of initiation to cannabis use, driving after recent cannabis use, and riding with a driver who had recently used cannabis. Of note, in 2023–24, question wording changed from “used marijuana or cannabis” to “smoked or vaped cannabis” for both motor vehicle questions. Variables were assessed beginning in 2014–15 unless stated otherwise in Results. Detailed measures are available in Supplementary Materials.

### 2.2 Data analysis

Changes over time in binary outcomes were analyzed using logistic regression. Survey cycle was entered as a categorical variable in all models; regression results refer to changes since 2014–15 or the cycle in which the variable was added. Models were additionally stratified by sex (male vs. female) and grade group (grade 7–9 vs. 10–12). Students below the legal driving age were excluded from the model on driving after cannabis use.^4^ Age of initiation was analyzed using linear regression and stratified by sex and grade group. Responses of not stated were excluded from regression models. Estimates were weighted using sampling weights to be representative of the entire student population in the provinces, as well as 500 averaged bootstrapped survey weights to account for the complex survey design and non-response. Data were analyzed using SVY procedures in STATA v.17.

## 3 Results

Table 1 shows sample characteristics for the 5 survey cycles. Mean participant age was 14.8 years (grades 7–9 =13.3 years; grades 10–12 =16.3 years) and 48.8 % were female.

**Table 1:** Sample characteristics, grade 7–12 students in the Canadian Student Alcohol and Drugs Survey 2014–15 to 2023-24 (weighted estimate, unweighted n) ^a^.

**Characteristic**	**Total** (n = 264,558)	**2014–15** (n = 36,665)	**2016–17** (n = 52,103)	**2018–19** (n = 62,850)	**2021–22** (n = 61,096)	**2023–24** (n = 51,844)
**Age** (mean years)^b^	14.8 (261,936)	14.7 (36,501)	14.7 (51,854)	14.7 (62,445)	14.8 (60,711)	14.8 (50,425)
**Sex** (%)						
Female	48.8 (132,033)	48.6 (18,590)	48.7 (26,141)	49.0 (31,066)	49.1 (30,451)	48.7 (25,785)
Male	51.2 (132,525)	51.4 (18,075)	51.3 (25,962)	51.0 (31,784)	50.9 (30,645)	51.3 (26,059)
**Grade group** (%)						
7–9	49.8 (145,290)	49.1 (19,215)	49.7 (28,831)	50.5 (36,375)	50.7 (35,991)	48.8 (24,878)
10–12	50.2 (119,268)	50.9 (17,450)	50.3 (23,272)	49.5 (26,475)	49.3 (25,105)	51.2 (26,966)
**Province** (%)^c^						
Newfoundland & Labrador	1.3 (25,950)	1.3 (3169)	1.4 (6045)	1.3 (5321)	1.2 (7032)	1.5 (4383)
Prince Edward Island	0.5 (20,308)	0.5 (1958)	0.4 (4536)	0.5 (4754)	0.5 (4616)	0.6 (4444)
Nova Scotia	2.7 (29,299)	2.6 (3868)	2.6 (4819)	2.5 (6470)	2.6 (6999)	3.1 (7143)
New Brunswick	1.4 (8522)	2.2 (307)	n/a	2.1 (3477)	n/a	2.6 (4738)
Quebec	15.1 (34,133)	18.3 (4183)	18.2 (3244)	18.3 (15,843)	18.3 (10,863)	n/a
Ontario	44.8 (33,701)	44.4 (5253)	44.9 (10,195)	42.7 (6063)	43.1 (6745)	49.6 (5445)
Manitoba	4.1 (21,783)	3.9 (3682)	4.1 (3864)	4.0 (3518)	4.0 (3100)	4.8 (7619)
Saskatchewan	3.4 (22,392)	3.1 (3357)	3.3 (3417)	3.2 (3868)	3.3 (5596)	4.1 (6154)
Alberta	12.9 (36,929)	11.1 (5547)	12.0 (9448)	12.3 (6318)	13.3 (9260)	16.4 (6356)
British Columbia	13.8 (31,541)	12.6 (5341)	13.1 (6535)	13.0 (7218)	13.8 (6885)	17.4 (5562)

^a^ Difference between survey cycles tested using linear regression for mean age, and Pearson chi-square test for proportions. Differences across survey cycles were significant for age, sex, grade group and province (p < 0.001 for all). ^b^ Mean age is not included in analyses and is included in sample table for descriptive purposes only. ^c^ New Brunswick did not participate in 2016–17 or 2021–22 and Quebec did not participate in 2023–24.

### 3.1 Age of initiation

As shown in Table 2, mean age of initiation to cannabis use did not change from 2014–15 to 2023–24 among grade 7–12 students (14.2 years; p = 0.421). It also did not change among grade 10–12 students (range=14.5–14.6 years; p = 0.201). However, grade 7–9 students started using cannabis at a significantly younger age over time, with an average of 13.0 years in 2014–15 versus 12.4 years in 2023–24 (p < 0.001). Stratified by sex, there was no change among males (range=14.1–14.2 years; p = 0.258). Females started using cannabis at a slightly (but significantly) younger age over time, with an average of 14.3 years in 2014–15 versus 14.1 years in 2023–24 (p < 0.001).

**Table 2:** Prevalence of cannabis use among students in grades 7–12 in the Canadian Student Alcohol and Drugs Survey 2014–15 to 2023-24.

	**2014–15** (n = 36,665)	**2016–17** (n = 52,103)	**2018–19** (n = 62,850)	**2021–22** (n = 61,096)	**2023–24** (n = 51,844)	**Change over time**^a^
	**Weighted % (95 % CI)**					
**Past 12-month cannabis use** (all respondents)						
Overall	16.5 (15.3–17.8)	16.7 (15.5–17.9)	18.1 (16.3–19.8)	18.3 (15.8–20.9)	18.3 (16.0–20.8)	F(4496)= 1.03, p = 0.391
Grade 7–9	5.7 (4.6–7.1)	5.5 (5.0–6.2)	7.0 (6.2–7.8)	7.9 (6.7–9.2)	6.8 (5.8–8.0)	F(4496)= 4.44, p = **0.002**
Grade 10–12	26.8 (25.0–28.6)	27.8 (25.6–30.0)	29.4 (26.3–32.6)	29.1 (24.9–33.6)	29.3 (25.3–33.5)	F(4496)= 0.74, p = 0.562
Female	16.2 (14.8–17.8)	15.8 (14.5–17.1)	18.1 (16.0–20.3)	19.7 (17.1–22.6)	19.8 (16.5–23.6)	F(4496)= 3.05, p = **0.017**
Male	16.8 (15.3–18.4)	17.6 (16.3–18.9)	18.0 (16.0–20.3)	16.9 (14.3–19.8)	16.8 (14.9–18.9)	F(4496)= 0.34, p = 0.848
**Past 30-day cannabis use** (all respondents)						
Overall	11.1 (10.2–12.1)	10.9 (10.0–11.8)	11.5^+^ (10.2–12.9)	12.6 (10.8–14.6)	11.9 (10.3–13.8)	F(4496)= 0.80, p = 0.552
Grade 7–9	4.1 (3.3–5.1)	3.7 (3.3–4.2)	4.3 (3.7–4.9)	5.8 (4.8–7.0)	4.7 (4.0–5.6)	F(4496)= 4.42, p = **0.002**
Grade 10–12	17.8 (16.4–19.2)	18.0 (16.4–19.8)	18.8 (16.5–21.4)	19.6 (16.7–22.8)	18.9 (15.9–22.3)	F(4496)= 0.40, p = 0.806
Female	10.4 (9.3–11.6)	9.4 (8.5–10.4)	11.0 (9.5–12.7)	13.4 (11.4–15.6)	12.5^↓^ (10.5–14.8)	F(4496)= 4.39, p = **0.002**
Male	11.8 (10.6–13.2)	12.3 (11.2–13.5)	11.9 (10.2–13.9)	11.8 (9.9–14.0)	11.5^↓^ (9.8–13.4)	F(4496)= 0.19, p = 0.944
**Frequent cannabis use**^**b**^**in past 30 days** (all respondents)						
Overall	5.6 (4.9–6.4)	5.6 (5.0–6.4)	5.4 (4.8–6.2)	7.1 (5.9–8.5)	6.6 (5.4–8.0)	F(4496)= 1.79, p = 0.129
Grade 7–9	2.0 (1.4–2.9)^M^	1.7 (1.5–2.0)	1.7 (1.5–2.0)	3.2 (2.5–4.1)	2.5 (2.0–3.2)	F(4496)= 6.45, p**< 0.001**
Grade 10–12	9.0 (8.0–10.1)	9.5 (8.3–11.0)	9.2 (8.0–10.6)	11.1 (9.2–13.4)	10.5^↓^ (8.4–12.9)	F(4496)= 1.07, p = 0.369
Female	4.6 (3.8–5.6)	3.9 (3.4–4.6)	4.4 (3.7–5.3)	7.0 (5.8–8.5)	6.2 (5.2–7.5)	F(4496)= 6.92, p**< 0.001**
Male	6.5 (5.7–7.5)	7.3 (6.3–8.3)	6.4 (5.4–7.4)	7.2 (5.8–8.9)	6.9 (5.5–8.7)	F(4496)= 0.50, p = 0.739
**Age of initiation to cannabis use** (students who have ever tried cannabis)	**2014–15** (n = 7405)	**2016–17** (n = 10,351)	**2018–19** (n = 13,159)	**2021–22** (n = 12,211)	**2023–24** (n = 12,245)	**Change over time**^a^
Overall	14.2 (14.1–14.3)	14.2 (14.2–14.3)	14.3 (14.1–14.4)	14.1 (14.0–14.2)	14.2 (14.1–14.3)	F(4496)= 0.97, p = 0.421
Grade 7–9	13.0 (12.7–13.2)	12.9 (12.8–13.0)	12.8 (12.7–12.9)	12.6 (12.5–12.7)	12.4 (12.2–12.5)	F(4496)= 10.30, p**< 0.001**
Grade 10–12	14.5^↓^ (14.3–14.6)	14.5 (14.4–14.6)	14.7 (14.5–14.8)	14.6 (14.5–14.7)	14.6 (14.5–14.7)	F(4496)= 1.50, p**=** 0.201
Female	14.3 (14.2–14.5)	14.4 (14.3–14.5)	14.4 (14.2–14.6)	14.2 (14.0–14.3)	14.1 (14.0–14.2)	F(4496)= 5.43, p**< 0.001**
Male	14.1 (13.9–14.2)	14.1 (14.0–14.2)	14.2 (14.1–14.3)	14.1 (14.0–14.2)	14.2 (14.1–14.4)	F(4496)= 1.33, p = 0.258

95 %CI= 95 % confidence interval. ^a^ Main effect of time (i.e., survey cycle) from logistic regression model testing change in estimate across 5 survey cycles (adjusted Wald test); bolded values are significant at p < 0.05. Rows for grade group and sex represent models stratified by each grade group or sex. ^b^ Frequent use was defined as using cannabis at least once per week in the past 30 days. ^M^ Marginal data quality (CV 16.6–33.3); interpret estimate with caution. ^↓^ Estimate should be rounded down if reported as a whole number.

### 3.2 Prevalence of cannabis use

Overall, there was no significant change in prevalence of past 12-month, past 30-day, or frequent use over the 10-year period. However, there were significant increases in all three prevalence indicators for females and grade 7–9 students (Table 2). In sensitivity analyses, provinces that did not participate in all survey cycles (New Brunswick and Quebec) were removed to ensure that inconsistent geographic coverage did not influence prevalence rates. The pattern of results was the same; therefore, all provinces were retained the sample.

### 3.3 Method of use (added in 2016–17)

In 2023–24, vaping was the most common method of cannabis use among all students in grade 7–12, with 16.4 % reporting vaping any form of cannabis in the past 12 months (including 15.5 %, 8.2 % and 6.3 % who vaped liquid, dried and solid cannabis, respectively).^5^ Smoking (14.8 %) and eating (10.3 %) cannabis were the second and third most common methods of use, respectively.

**Fig. 1:**
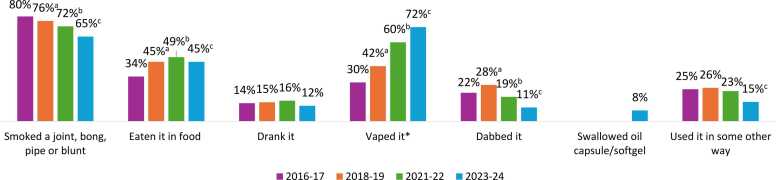
Methods of cannabis use among grade 7–12 students who used cannabis in the past 12 months, Canadian Student Alcohol and Drugs Survey, 2016–17 to 2023–24. ^⁎^Note that “vaped it” represents vaping cannabis in 2016–17 and 2018–19; vaping dried and/or liquid cannabis in 2021–22; and vaping dried, liquid and/or solid cannabis in 2023–24. ^a^ Significant difference between 2016–17 and 2018–19. ^b^ Significant difference between 2016–17 and 2021–22. ^c^ Significant difference between 2016–17 and 2023–24 (p < 0.05).

**Fig. 2:**
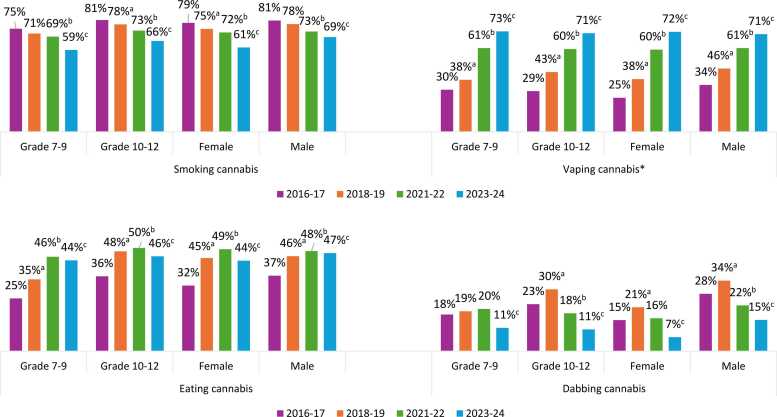
Methods of cannabis use, stratified by grade group and sex, among grade 7–12 students who used cannabis in the past 12 months, Canadian Student Alcohol and Drugs Survey, 2016–17 to 2023–24. ^⁎^Note that “vaping cannabis” represents vaping cannabis in 2016–17 and 2018–19; vaping dried and/or liquid cannabis in 2021–22; and vaping dried, liquid and/or solid cannabis in 2023–24. ^a^ Significant difference between 2016–17 and 2018–19. ^b^ Significant difference between 2016–17 and 2021–22. ^c^ Significant difference between 2016–17 and 2023–24 (p < 0.05).

Fig. 1 shows methods of cannabis use among students who had consumed cannabis in the past 12 months, and Fig. 2 shows grade and sex stratification for the most common methods of use. Among students who consumed in the past year, smoking remained the most common method of cannabis use until 2021–22 but declined significantly over the study period in all grade and sex groups (p < 0.001 for all), with the largest decrease in females. Vaping increased significantly over the study period in all grade and sex groups (p < 0.001 for all) and was reported by over 7 in 10 students who used cannabis in 2023–24. The largest increase was observed in females, with a quarter of females who consumed cannabis reporting vaping cannabis in 2016–17 and almost three quarters in 2023–24.

Consuming edibles increased since 2016–17 in all grade and sex groups (p < 0.001 for all), with the largest increase in grade 7–9 students, and a plateau in all groups in recent cycles. Dabbing decreased by half over the study period, with significant decreases in all grade and sex groups (p ≤ 0.01 for all), and the largest decreases observed in males and grade 10–12 students. There was no clear trend for drinking cannabis beverages except for a decrease from 2021–22 to 2023–24 (p = 0.017 for main effect).

### 3.4 Usual cannabis source (added in 2016–17)

**Fig. 3:**
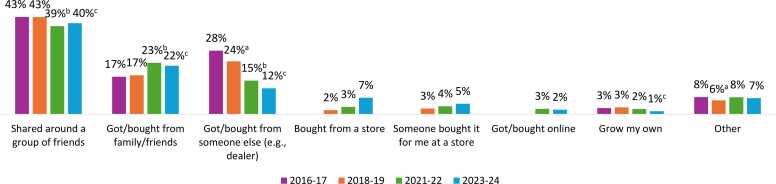
Usual source of cannabis among grade 7–12 students who used cannabis in the past 12 months, Canadian Student Alcohol and Drugs Survey, 2016–17 to 2023–24. ^+^ Response options for usual cannabis source are shown for survey cycles in which they appeared. Taking cannabis from a family member/friend without permission (range 0.9 %-2.9 %) and taking cannabis from someone else without permission (0.3 %–0.7 %) were uncommon and were excluded from the figure. For usual source, estimates were compared to 2014–15: ^a^ Significant difference between 2016–17 and 2018–19. ^b^ Significant difference between 2016–17 and 2021–22. ^c^ Significant difference between 2016–17 and 2023–24 (p < 0.05).

Fig. 3 shows the ‘usual source’ of cannabis used by students who had consumed cannabis in the past 12 months*.* The most common sources were social sources: shared around a group of friends (which decreased since 2016–17) and got/bought from a family member/friend (which increased since 2016–17) (p ≤ 0.01 for both); both sources plateaued in the most recent cycle. There were clear changes over time in all grade and sex groups for getting/buying from someone else (e.g., dealer), which became less prevalent over time (p < 0.001 for all); and buying from a store, which was relatively uncommon but became more prevalent over time (increased from 1.9 % in 2018–19–7.4 % in 2023–24) (p < 0.0001). This increase was observed in all sex and grade groups (p < 0.05 for all), with a larger increase in grade 10–12 students (305 % growth rate) than grade 7–9 students (200 % growth rate); data not shown. The remaining sources were used by < 10 % of students who consumed cannabis.

### 3.5 Ease of access to cannabis

The proportion of grade 7–12 students reporting it was “fairly” or “very” easy to access cannabis increased from 2014–15 to 2023–24 (41.4–49.2 %; p < 0.001), with the increase driven by the most recent cycle. This increase was observed in males, females, and grade 7–9 students (p < 0.001 for all). The belief that “it has been easier” to access cannabis since legalization also increased from 2018–19 to 2023–24 (3.7–8.2 %) and was observed in all grade and sex groups (p < 0.001 for all).

### 3.6 Perceived risk of regularly consuming cannabis

In all years, the majority of grade 7–12 students felt there was a “great” or “moderate” risk of regularly smoking cannabis (declining from 76.3 % in 2014–15 to 72.2 % in 2023–24). Perceived risk of regularly smoking cannabis decreased over time in males, females, and grade 7–9 students (p < 0.001 for all). In the 2023–24 survey, which also asked about risk of vaping and eating cannabis, perceived great/moderate risk was slightly higher for vaping cannabis (74.0 %) and smoking cannabis (72.2 %) than for eating cannabis (65.7 %).

### 3.7 Beliefs about specific health effects of cannabis (added in 2023–24)

When asked, “How much do you think people are at risk of the following when using cannabis on a regular basis?”, most grade 7–12 students acknowledged a “great” or “moderate” risk of “developing an addiction to cannabis” (82.1 %) and “harming ability to remember or pay attention” (76.5 %). Fewer acknowledged a great/moderate risk of “developing or worsening anxiety or depression” (68.9 %). Similarly, regarding the proportion selecting “no” or a “slight” risk, fewer acknowledged the risk to mental health: 1 in 5 students (20.5 %) felt that cannabis posed little risk of developing or worsening anxiety or depression, compared to 13.0 % for harming one’s ability to remember or pay attention and 10.5 % for developing addiction. For each health effect, a sizeable proportion (7.3 %-10.6 %) responded that they did not know (approximately 126,000–183,000 students).

### 3.8 Motor vehicle behaviours

**Fig. 4:**
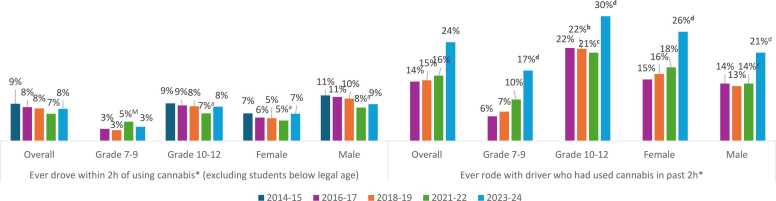
Motor vehicle behaviours among grade 7–12 students in the Canadian Student Alcohol and Drugs Survey, 2014–15 to 2023–24. ^⁎^Note that in 2023–24, question wording for both driving and passenger behaviours was changed from “used marijuana or cannabis” to “smoked/vaped cannabis”. ^M^ Estimate for grade 7–9 in 2021–22 associated with moderate sampling variability; interpret with caution. Estimate for grade 7–9 in 2014–15 is suppressed due to high sampling variability. For driving behaviours, estimates were compared to 2014–15: ^**a**^ Significant difference between 2014–15 and 2021–22. For passenger behaviours, estimates were compared to 2016–17 (year question was added): ^**b**^ Significant difference between 2016–17 and 2018–19. ^**c**^ Significant difference between 2016–17 and 2021–22. ^**d**^ Significant difference between 2016–17 and 2023–24.

As shown in Fig. 4, there was very little overall change in the prevalence of ever driving after using cannabis among students of legal driving age, with estimates returning to 2014–15 levels after a significant decrease in 2021–22 (p = 0.042 for main effect). There was also no significant change in driving after cannabis use in models stratified by sex or grade group, with the only significant changes occurring in 2021–22. Rates of ever riding with a driver who had used cannabis increased significantly from 2016–17 to 2023–24 (p < 0.001), driven largely by the most recent cycle. This increase was observed in all grade and sex groups (p < 0.001 for all), with 3 in 10 grade 10–12 students reporting this behaviour in 2023–24 (Fig. 4). As shown online, a parallel increase was not observed for driving after alcohol use or riding with a driver who had consumed alcohol (Health Canada, 2025c).

## 4 Discussion

The current study provides estimates of cannabis use among a large, population-based sample of youth in the Canadian provinces over a 10-year period. Rates of cannabis use among grade 7–12 students in the most recent cycle of CSADS generally aligned with rates among youth^6^ in large Canadian and US surveys (within 5 %) (CDC, 2021; Centre for Addiction and Mental Health (CAMH), 2023a, Centre for Addiction and Mental Health (CAMH), 2023b, Centre for Addiction and Mental Health (CAMH), 2024). There were, and are, expressed concerns about cannabis legalization and the impact on youth cannabis use and the safety of youth (Ireland, 2018, Porter, 2018). Study results suggest that overall rates of youth cannabis use have not changed substantially since before cannabis legalization. Similarly, the Ontario Student Drug Use and Health Survey (OSDUHS) reported decreasing youth cannabis use from 1999 to 2023, and CCS found an initial increase among youth aged 16–19 immediately after legalization, followed by a plateau (CAMH, 2023b; Government of Canada, 2024a). While the overall findings suggest some pre-legalization concerns have not materialized, it is notable that despite a series of federal and provincial measures to protect youth, some key indicators have not improved and rates have increased in some subgroups, as discussed below.

Restricting access to cannabis is a key youth protection measure. A recent study analyzed CSADS data and reported an increase in past 12-month cannabis use among grade 7–11 students between 2018–19 and 2021–22 (Mital and Nguyen, 2025). The current study examined grade 7–12 students and found no change in past 12-month cannabis use overall; however, the present analysis of five survey cycles demonstrated small but significant increases in past 12-month, past 30-day, and frequent use over time in grade 7–9 students (representing 18 %, 15 %, and 25 % growth rates, respectively). Similar to 2023 OSDUHS (CAMH, 2023b), cannabis use increased with grade, with substantially higher rates among grade 10–12 than 7–9 students. Moreover, students perceived cannabis as becoming easier to access over time, and obtaining cannabis from retail stores increased significantly since 2018–19, despite most respondents being below the legal purchase age. Addressing perceived access to cannabis may be challenging given legalization and the ubiquity of legal retail stores across Canada (Statistics Canada, 2023). However, given that cannabis use during youth and young adulthood can harm the developing brain (CDC, 2024), increases in cannabis use and decreasing age of initiation in younger students and greater perceived access overall are concerning and suggest a potential increase in risk of harm associated with cannabis use without continued education and prevention measures.

The results also speak to a broader trend in which rates of cannabis use in females and girls/women are increasing to match or exceed those of males and boys/men, who traditionally had higher rates of use. This has been observed in recent surveys among adults in Canada (CAMH, 2023a; Government of Canada, 2017, Government of Canada, 2024c) and among youth in both the US (Johnson et al., 2015) and Canada (Centre for Addiction and Mental Health (CAMH), 2017, Centre for Addiction and Mental Health (CAMH), 2019, Centre for Addiction and Mental Health (CAMH), 2022, Centre for Addiction and Mental Health (CAMH), 2023b; University of Waterloo, 2025b; WHO, 2023). This trend may reflect shifting social norms, with the liberalization of cannabis laws in North America (Pew Research Center, 2024) translating to less stigma surrounding cannabis use in both sexes. Increased rates of use in young females also may be attributable to poor mental health. Examining interactions between mental health, sex and cannabis use was beyond the scope of this study. However, CSADS data show that in 2023–24, past 12-month cannabis use was approximately 16 % higher among students with poor/fair self-rated mental health compared to those with better mental health (Health Canada, 2025c). In addition, 2023 OSDUHS found that compared to male students, females reported poorer mental health, lower self-esteem and more stress, and were more likely to use cannabis to cope with mental health concerns (CAMH, 2024). Frequent, prolonged cannabis use during adolescence or young adulthood may lead to worsening mental health, including anxiety, depression, psychosis, and suicidal ideation and attempt (Bagot et al., 2015, Bataineh et al., 2023, Gobbi et al., 2019, Sultan et al., 2023). Given that young people may be using cannabis as a coping mechanism for mental health issues but also may experience poorer mental health as a result of cannabis use, there is a need for increased education and supports for young people struggling with mental health challenges and substance use. This may be especially true for females who use cannabis, who have been shown to progress to cannabis use disorder more quickly than males (Towers et al., 2023).

Regarding methods of cannabis use, vaping surpassed smoking in 2023–24, increasing at growth rates of 193 % and 108 % among females and males, respectively, over the study period. Other studies have similarly reported smoking and vaping as the two most common methods of use, with increased rates of youth vaping over time (CAMH, 2023b; Government of Canada, 2017, Government of Canada, 2024c; Health Canada, 2025a; Lim et al., 2022). A recent secondary analysis of CSADS data (Mital and Nguyen, 2025) found that vaping increased to a similar extent in Quebec (where vape liquids are not available from legal retailers, and inhalable cannabis products are capped at 30 % THC) (Gouvernement du Québec, 2025) and the remaining Canadian provinces. This suggests that vaping is pervasive among students, even in jurisdictions where vape liquids are not available at legal retail outlets. While different forms of cannabis can be vaped, vape liquids were the predominant form reported in CSADS and two other Canadian surveys (Government of Canada 2024b, Health Canada, 2025a). This is concerning, since most cannabis vape liquids sold in Canada are very potent (with THC concentrations reaching 90 %-98 %) (BC Cannabis Stores, 2025, Ontario Cannabis Store, 2025), and high THC concentrations can lead to greater risk of dependence and adverse effects (Government of Canada, 2021b). Research suggests that youth vape for various reasons, including to control dosage, because they perceive it as trendy, enjoyable, and discreet, and as a way to reduce stress and elevate mood (Harrell et al., 2022). Given the increasing popularity of vaping over time and these apparent motivations for continued use, curbing cannabis vaping in young people (especially females) may be challenging, but efforts are warranted.

Another key measure to address youth cannabis use is public education, including communicating accurate information to Canadian youth about the risks posed by cannabis products. Almost three-quarters of grade 7–12 students in 2023–24 CSADS reported that both smoking and vaping cannabis carried a great/moderate risk of harm, and almost two-thirds attributed this level of risk to edibles, mirroring results for youth in 2024 CCS (Government of Canada, 2024c). This suggests youth are aware of potential harms, although additional factors may contribute to an individual’s decision to use cannabis. Efforts to prevent or delay cannabis use initiation are important as early initiation to cannabis use and frequent use can increase one’s risk of cannabis dependence and impair brain function (Government of Canada, 2024d). Reassuringly, age of initiation remained constant over the study period. However, our findings suggest students may lack a detailed understanding of specific risks related to mental health, addiction, and cognition; additionally, perceived risk of regularly smoking cannabis declined over time. Education campaigns may play a role in elevating and maintaining risk perceptions of regular cannabis use, as evidenced by a recent Canadian study (Schwartz et al., 2024). Since legalization, Health Canada has conducted several cannabis education campaigns aimed at youth, including a novel school-based campaign aiming at increasing adolescents’ awareness of the effects of cannabis use (Health Canada, 2025e). Continued public education programs can help maintain and improve youth awareness of the potential harms of cannabis, as part of a multi-pronged strategy to mitigate increasing rates of cannabis use, especially vaping, among younger students and females.

### 4.1 Strengths and limitations

This study was strengthened by its rigorous methodology, sampling design and survey weights, with estimates drawn from a large, representative sample of the entire student population in the Canadian provinces. The study also benefited from having five data points spanning a decade that included pre-, peri- and post-legalization time points. Future survey cycles will provide more data with which to determine detailed trends, especially for indicators added in recent years. The study was subject to standard limitations of survey data, including recall and social desirability bias. However, some bias may have been attenuated by the school-based setting, since previous research suggests that many youth feel more comfortable admitting to drug use at school versus home, and consider a school setting to be more confidential (United Nations Office on Drugs and Crime, 2003). Finally, provincial analyses were not presented despite differing retail frameworks among the provinces; these have been recently examined elsewhere (Mital and Nguyen, 2025).

## 5 Conclusion

This study bolstered previous efforts to examine youth cannabis use in Canada from a large sample of almost 265,000 students by providing detailed estimates over a period that spanned the legalization and regulation of cannabis for non-medical purposes. Results suggest that while overall rates of cannabis use remained fairly stable, certain trends are concerning and should continue to be closely monitored, including increasing cannabis use and decreasing age of initiation among younger students and females, decreasing risk perceptions, increased perceived ease of access, and the growing popularity of high-potency products such as vape liquids.

## Authors’ contribution and statement

SG: Formal analysis; Writing – Original Draft; SS: Investigation; Writing – Review & Editing; EK: Investigation; Writing – Review & Editing; HA: Writing – Review & Editing; Supervision. The content and views expressed in this article are those of the authors and do not necessarily reflect those of the Government of Canada.

## Funding

Funding for this project was provided by 10.13039/501100000008Health Canada.

## Declaration of Competing Interest

Samantha Goodman: I have nothing to declare.

Sebastian Srugo: I have nothing to declare.

Emilia Krzeminska: I have nothing to declare.

Hanan Abramovici: I have nothing to declare.

## Data Availability

Canadian Student Alcohol and Drugs Survey Public Use Microdata Files (PUMFs) are available online at: https://open.canada.ca/data/en/dataset/1f15ca45-8bfd-4f9c-9ec6-2c0c440e69c2

## Footnotes

1 Herein, ‘cannabis legalization’ will refer to legalization and regulation of cannabis for non-medical purposes in Canada.

2 In the province of Quebec, students are in Secondary I-V, corresponding to grades 7–11. Note that the 2014–15 cycle included grade 6 students who were not asked drug-use questions and were excluded from the current analysis.

3 New Brunswick did not participate in 2016–17 and 2021–22 and Quebec did not participate in 2023–24.

4 The survey question also covers snowmobiles, motor boats, and all-terrain vehicles (ATVs) which may have lower legal age requirements than road vehicles. For simplicity, this analysis was limited to those above the legal age for road vehicles (14 years in Alberta, 16 years elsewhere).

5 Responses were not mutually exclusive; some students vaped more than one form of cannabis.

6 Since each survey measures youth differently, for the purposes of this Discussion, ‘youth’ refers to grade 7–12 students in OSDUHS, 12–19-year-olds in CCHS, 15-year-olds in HBSC and 16–19-year-olds in CCS.
