# Supplementary material for: Changes in patterns of use and perceptions of cannabis among students in Canada: A decade of data from the Canadian Student Alcohol and Drugs Survey
Source: Drug Alcohol Depend Rep. 2025 Nov 25;17:100399. doi: 10.1016/j.dadr.2025.100399 (PMC12712680; doi:10.1016/j.dadr.2025.100399)
Supplement: Supplementary file 1 — Supplementary material [file mmc1.docx]

**Supplemental File 1**

**Measures**

The following CSADS cannabis questions were included in the analysis. Unless otherwise noted, questions were asked since 2014/15.

| **Measure** | **Variable coding** |
| --- | --- |
| **Age of initiation:** “How old were you when you first used cannabis?”   - 8 years or younger^7^ - 9 years - 10 years - 11 years - 12 years - 13 years - 14 years - 15 years - 16 years - 17 years - 18 years or older | Continuous variable (valid range: 8 to 18 years)  ^7^8 years or younger recoded to 8 years; 18 years or older recoded to 18 years |
| **Past 12-month cannabis use:** “In the last 12 months, how often did you use cannabis?”   - I have not used cannabis in the last 12 months - Less than once a month - Once a month - 2 or 3 times a month - Once a week - 2 or 3 times a week - 4 to 6 times a week - Every day | 1=Yes, in the past 12 months  0=No |
| **Current (past 30-day) cannabis use:** “In the last 30 days, how often did you use cannabis?”   - I have not used cannabis in the last 30 days - Once or twice - Once or twice a week - 3 or 4 times a week - 5 or 6 times a week - Every day | 1= Yes, in the past 30 days  0=No |
| **Frequent (at least weekly) use:** “In the last 30 days, how often did you use cannabis?”   - I have not used cannabis in the last 30 days - Once or twice - Once or twice a week - 3 or 4 times a week - 5 or 6 times a week - Every day | 1=At least once per week in past 30 days  0=Less frequently |
| **Method of cannabis use (added in 2016/17):** “Indicate whether you have used cannabis (a joint, pot, weed, hash, or hash oil) in the following ways”   1. Smoked a joint, bong, pipe or blunt 2. Eaten it in food such chocolate, gummies, brownies, or cookies 3. Drank it in sparkling water, iced tea, soft drinks (dissolvable powder) 4. Vaped dried cannabis^1^ (e.g., using the same type of cannabis used in a joint) **(added in 2021/22)** 5. Vaped liquid cannabis^1^ (e.g., in a vape pen/cartridge) **(added in 2021/22)** 6. Vaped solid cannabis^1^ (e.g., shatter, wax, hash, or kief in a vape pen) **(added in 2023/24)** 7. Dabbed it (using a hot knife or nail to heat solid extracts) 8. Swallowed an oil capsule or softgel **(added in 2023/24)** 9. Used it some other way  - **No**, I have never done this - **Yes**, I have done this in the last 30 days - **Yes**, I have done this in the last 12 months - **Yes**, I have done this, but **not** in the last 12 months | 1=Yes, in the past 12 months  0=No, never or not in past 12 months  ^1^Note: prior to 2021/22, there was only 1 item on vaping (‘Vaped cannabis’). To establish consistency across survey cycles, a binary variable was created where ‘Vaped cannabis’ included respondents who vaped cannabis in 2014/15 to 2018/2019, or who vaped dried, liquid, and/or solid cannabis in 2021/22 to 2023/24. |
| **Usual cannabis source (added in 2016/17**): “In the last 12 months, how did you usually get the cannabis you used?”   - I have not done this in the last 12 months - I grow my own - It was shared around a group of friends - I took it from a family member or friend without their permission - I took it from someone else without their permission - I got or bought it online (e.g., website, social media store, etc.) - I got or bought it from a family member or a friend - I got or bought it from someone else (e.g., a dealer) - I bought it from a store - Someone bought it for me at a retail store | 1=Yes, in the past 12 months  0=No |
| **Perceived risk:** “How much do you think people risk harming themselves when they do each of the following activities?”   1. Smoke cannabis on a regular basis^2^ 2. Vape cannabis on a regular basis ***(added in 2023/24)*** 3. Eat cannabis on a regular basis ***(added in 2023/24)***  - No risk - Slight risk - Moderate risk - Great risk - I do not know | 1=Moderate risk/Great risk  0=No risk/Slight risk/Don’t know  ^2^Other drugs were included in the list of response options but only cannabis was included in the current analysis. |
| **Beliefs about specific health effects of cannabis: (added in 2023/24):** “How much do you think people are at risk of the following when using cannabis on a regular basis?”   1. Developing or worsening anxiety or depression 2. Developing an addiction to cannabis 3. Harming their ability to remember or pay attention  - No risk - Slight risk - Moderate risk - Great risk - I do not know | 1=No risk/Slight risk  2=Moderate risk/Great risk  3=Don’t know |
| **Ease of access to cannabis:** “How difficult or easy do you think it would be for you to get each of the following types of substances, if you wanted some?”   1. Cannabis^3^  - Very difficult - Fairly difficult - Fairly easy - Very easy - I do not know | 1=Fairly easy/Very easy  0 Very difficult/fairly difficult/Don’t know  ^3^Other drugs were included in the list of response options but only cannabis was assessed in the current analysis. |
| **Ease of access to cannabis since legalization (added in 2018/19):** “The use of cannabis was made legal for adults in Canada in 2018. Has it been easier to get cannabis for yourself after legalization?”   - I have never bought/got cannabis - It has been easier - It has been harder - Neither easier nor harder | 1=It has been easier  0=I have never bought cannabis/It has been harder/Neither easier nor harder |
| **Driving after using cannabis:** “Have you ever driven a vehicle (e.g., car, snowmobile, motor boat, or all-terrain vehicle (ATV))…”   1. Within 2 hours of using marijuana or cannabis? ^4,5^  - **No**, never - **Yes**, in the last 30 days - **Yes**, more than 30 days ago | 1=Yes, ever  0=No, never  ^4^In 2023/24, wording was changed to ‘smoking or vaping cannabis’.  ^5^Drinking alcohol was included in the list of response options but only cannabis was included in the current analysis. |
| **Riding with a driver who has used cannabis:** “Have you ever been a passenger in a vehicle (e.g., car, snowmobile, motor boat, or all-terrain vehicle (ATV))…”   1. Within 2 hours of using marijuana or cannabis?^4,5^  - **No**, never - **Yes**, in the last 30 days - **Yes**, more than 30 days ago - **I do not know**^6^ | 1=Yes, ever  0=No, never  ^4^In 2023/24, wording was changed to ‘smoking or vaping cannabis’.  ^5^Drinking alcohol was included in the list of response options but only cannabis was included in the current analysis.  ^6^Those who responded ‘I do not know’ were excluded from the analysis. |
